# Study protocol: a mixed-methods study to evaluate which health visiting models in England are most promising for mitigating the harms of adverse childhood experiences

**DOI:** 10.1136/bmjopen-2022-066880

**Published:** 2022-09-28

**Authors:** Jenny Woodman, Louise Mc Grath-Lone, Amanda Clery, Helen Weatherly, Dina Jankovic, Jane V Appleton, Jennifer Kirman, Jane Barlow, Sally Kendall, Samantha Bennett, Ruth Gilbert, Katie Harron

**Affiliations:** 1 Social Research Institute, UCL-Faculty of Education and Society (IOE), London, UK; 2 Population, Policy and Practice, UCL Great Ormond Street Institute of Child, London, UK; 3 Centre for Health Economics, University of York, York, UK; 4 OxINMAHR (Oxford Institute of Nursing, Midwifery and Allied Health Research), Oxford Brookes University, Oxford, UK (Please note JVA is formerly of OxINMAHR but now retired); 5 Oxford School of Nursing and Midwifery, Oxford Brookes University, Oxford, UK; 6 Department of Social Policy and Intervention, University of Oxford, Oxford, UK; 7 Centre for Health Services Studies, University of Kent, Canterbury, UK; 8 Strategic Commissioning, Kent County Council, Maidstone, UK

**Keywords:** PUBLIC HEALTH, Child protection, QUALITATIVE RESEARCH, Community child health, Health policy, EPIDEMIOLOGY

## Abstract

**Introduction:**

Exposure to adverse childhood experiences (ACEs) is associated with poorer health outcomes throughout life. In England, health visiting is a long-standing, nationally implemented service that aims to prevent and mitigate the impact of adversity in early childhood, including for children exposed to ACEs. A range of health visiting service delivery practices exist across England (from the minimum five recommended contacts to tailored intensive interventions), but there is a lack of evidence on who receives what services, how this varies across local authorities (LAs) and the associated outcomes.

**Methods and analysis:**

This study will integrate findings from analysis of individual-level, deidentified administrative data related to hospital admissions (Hospital Episode Statistics (HES)) and health visiting contacts (Community Services Data Set (CSDS)), aggregate LA-level data, in-depth case studies in up to six LAs (including interviews with mothers), a national survey of health visiting services, and workshops with stakeholders and experts by experience. We will use an empirical-to-conceptual approach to develop a typology of health visiting service delivery in England, starting with a data-driven classification generated from latent class analysis of CSDS-HES data, which will be refined based on all other available qualitative and quantitative data. We will then evaluate which models of health visiting are most promising for mitigating the impact of ACEs on child and maternal outcomes using CSDS-HES data for a cohort of children born on 1 April 2015 to 31 March 2019.

**Ethics and dissemination:**

The University College London Institute of Education Research Ethics Committee approved this study. Results will be submitted for publication in a peer-reviewed journal and summaries will be provided to key stakeholders including the funders, policy-makers, local commissioners and families.

**STRENGTHS AND LIMITATIONS OF THIS STUDY**

- Our integration of linked administrative data with survey data and in-depth interview data will quantify health visiting services for families with a history of maternal adversity as compared with other children in England while also explaining why health visiting delivery and impact might vary across different local areas and for different family contexts.
- We have access to individual-level CSDS (Community Services Data Set) for 1 April 2015 and 31 March 2019, which provides information on health visiting contacts (type, frequency, length and date) for all children in England (approx. 3 million), linked to mother–child paired records of hospital admissions in HES (Hospital Episodes Statistics), which allows us to identify our exposure (adverse childhood experiences, ACEs) independently of what is known to and recorded by the health visiting service.
- We will refine our data-driven approach to characterising health visiting in England (latent-class analysis of CSDS-HES) using a workshop with up to 40 professional stakeholders, 4 workshops with experts by experience including one with fathers and in-depth qualitative interviews with professionals and parents in up to six local areas of England.
- As there are high levels of incompleteness in the CSDS we will use a ‘research-ready’ subset of sufficiently complete data which we estimate will capture health visiting for children living in approximately 25% of the 152 local areas in England and conduct a survey to gather information about local areas not contributing data to the research-ready subset of CSDS and to gather information not included in CSDS.
- For methodological reasons, this study only focuses on a subset of ACEs (parental alcohol and substance misuse, parental mental health problems and domestic violence and abuse between parents) as recorded in hospital admissions records for mothers, which represents about 38% of all ACEs known to health services (based on previous research).

## Introduction

Exposure to adverse childhood experiences (ACEs) is associated with a range of health-harming behaviours, and physical and mental health conditions in adolescence and mid-adulthood.^1^ Because ACEs are socially patterned, they also contribute to health inequalities that start in childhood and persist throughout adult life.^2 3^ In addition to the harm caused to individuals, ACEs place a large burden on public services and government spending, running into tens of billions of pounds annually.^4^

Health visiting is a long-standing, nationally implemented service that aims to prevent and mitigate the impact of adversity in early childhood and reduce the impact of inequalities in child development,^5^ including for children affected by ACEs. Health visitors lead the universal service for preschool children (aged <5 years) in England through the Healthy Child Programme (HCP),^6^ which is commissioned by local authorities (LAs). Health visitors review parent and child health and child development, and offer support in a range of areas, signposting to community resources such as children’s centres and state subsidised nurseries as appropriate.^7^ Some families are given extra help with feeding or sleeping while others (such as those with ACEs) have complex needs requiring a multiagency coordinated response.^5 7^ This model of ‘dialling up’ and ‘dialling down’ between universal and intensive services according to a continuous needs assessment has been described as ‘proportionate universalism’ or, in English policy documents a service that is ‘universal in reach—personalised in response’. and is at the heart of health visiting policy in England.^3 7^ In this model, health visitors provides four levels of service currently referred to in policy documents as community, universal, targeted and specialist.^8^

Health visiting policy mandates five contacts with every child and family in England (from 28 weeks pregnancy, 10–14 days and 6–8 weeks after birth, 9–12 months and 2–2.5 years) but also makes clear that ‘mandated reviews are not the full extent of the health visiting service offer’ and families may require additional contacts.^8^ An update to health visiting policy in 2021 suggests additional routine contacts at 3 and 6 months and refreshed the key high impact areas for health visiting services.^8^ (The six early years high impact areas outlined in the 2021 policy update are: (1) supporting the transition to parenthood; (2) supporting maternal and family mental health; (3) supporting breast feeding; (4) supporting healthy weight, healthy nutrition; (5) improving health literacy; reducing accidents and minor illnesses; (6) supporting health, wellbeing and development: ready to learn, narrowing the ‘word gap’. A key argument for universal and repeated contacts is that they provide opportunities for health visitors to identify families who need extra support.^9^ Frequent contacts also allow health visitors to develop relationships and trust with parents that are essential for the relational aspect of health visiting, in which parents are supported, guided, and advised to negotiate the journey into and through parenthood, thereby building self-efficacy, capacity and competence.^10 11^ Types of health visiting contact can include home visits, individual or group clinic appointments, or phone calls.^7^ English policy has now reverted to precovid guidance: all mandated reviews should be in-person (not telephone or video).^8 12^ Qualitative studies suggest that health visitors and parents agree that home visits rather than contacts in a clinic are best in terms of providing support.^10 11^

The importance of the intensity of home visits (ie, patterns of repeat contact) for helping the most vulnerable families underpins specialist programmes such as the family nurse partnership (FNP),^13^ in which specially trained family nurses, some of whom are health visitors, visit young first time mothers up to 64 times before the child’s second birthday.^11^ FNP is an evidence-based intervention developed in the USA, which theorises that frequent contact with family nurses can mitigate the impact of adversity by improving parental access to support services, and by increasing warm, sensitive and competent parenting and parental self-efficacy. This can positively impact the quality of caregiving and disrupt learnt behaviours of coercive control and negative parenting which can improve the quality of a child’s attachment to their primary caregivers, the child’s development and behaviour, and child safety and health.^14^

In terms of health visiting service delivery, a range of practices (from the minimum five recommended contacts to the intensive FNP offer) exist across England, but we lack evidence on who received what and how this varied across LAs. For example, despite the theorised importance, we know little about the intensity and type of health visiting contacts families receive, including those exposed to ACEs. At a local level, health visiting commissioners have been making difficult decisions about how to use scarce resources, with considerable variation in local need and service context (eg, the closure of Children’s Centres).^15^ Some LAs have responded to shrinking budgets, insufficient workforce and increased need in their population by using less qualified professionals, clinic instead of home visits, and group instead of individual sessions, but without evidence to underpin such resource-use decisions.^16^ LAs have also been making decisions about whether to focus limited resources on universal or targeted services, without evidence on the coverage of services for those most in need. Postpandemic, decisions will need to be made about how health visitors can best support children exposed to ACEs to recover from the secondary effects of COVID-19, such as unemployment, debt and missed early years education. At a national level, the organisation of the HCP and commissioning structure of health visiting is also currently under review.^17^ However, these decisions and policy reviews are occurring in the absence of an evidence-base about ‘business-as-usual’ health visiting,^18 19^ including the impact of different intensities or types of contact.

This study will develop a typology of health visiting service delivery models based on analysis of quantitative and qualitative data. Typologies are useful for describing and making sense of complex health and social care services whose delivery can vary across local areas.^20–22^ We will describe the different models of health visiting service delivery in England, including how and why models vary across LAs. Recent methodological developments have enabled linkage of routinely collected hospital admissions data and health visiting data which allows the association between health visiting contacts and outcomes to be examined at a whole population-level for the first time.^23^ This study will focus on a subset of ACEs (parental alcohol and substance misuse, parental mental health problems and domestic violence and abuse (DVA) between parents), which can compromise safe and nurturing home environments and hinder secure parent–child relationships,^1^ and are relatively common. For example, more than 10% of children live with an adult who misuses substances, 4% live with parents dependent on alcohol or substances and 20% live with parents who have high-risk alcohol use.^24 25^ Self-report studies show that around 32% of children live with a parent who has moderate or severe mental health problems and 7% of adults with children have experienced DVA in the last year.^24^ These ACEs can also be identified in hospital administrative data of mothers linked to the child’s record. These cases reflect a subset of mothers: of all mothers who give birth in hospital between 2010 and 2015 in England, 4.2% had a hospital admission for mental health or behavioural issues in the 2 years before the birth and 2.7% had an admission for ‘adversity’ (substance misuse, self-harm and/or violence).^26^ We will map different ways of delivering health visiting (eg, balance between targeted and intense health visiting for families exposed to ACEs and all families or for example, differences in use of home vs clinic contacts) onto geographical areas. This will allow us to evaluate which models of health visiting identified in our typology are most promising for mitigating the impact of these ACEs on emergency use of hospital services by children and their mothers (table 1).

**Table 1:** Evaluation outcomes for different models of health visiting service delivery

Domain	Outcome	Data source
Child development	Ages and Stages questionnaire scores at 2–2.5 years	CSDS
Child safety	Repeated A&E admissions up to age 5 years	HES
	Unplanned hospital admissions for injuries up to age 5 years	HES
	Unplanned, maltreatment-related hospital admissions up to age 5 years	HES
	Mortality up to age 5 years	HES
Exposure to ACEs	Mother’s hospital admissions for mental health conditions, substance misuse or violence up to 5 years after child’s birth	HES

ACEs, adverse childhood experiences; A&E, Accident & Emergency; CSDS, Community Services Data Set; HES, Hospital Episodes Statistics.

Our approach to evaluating the impact of health visiting for families with children exposed to ACEs has been driven by inherent difficulties in establishing a comparison group and a need to understand the high level of variation in health visiting across the country. health visitors has been a statutory universal service in England since 1929, which means that we do not have a comparison group of families who have never received health visiting. Nor can we compare outcomes associated with differing numbers of types (eg, home visiting vs clinical contact) of health visiting, among families with children exposed to ACEs. This is because the intensity and type of health visitors is likely to be driven by a particular family’s need, which we cannot robustly measure or account for in our data. For example, families with the most need for support should receive most health visiting, but these families will also be at risk of worse outcomes. Given that we do not have the data to match families on indicators of need, this analysis could generate spurious results (more frequent health visiting contacts associated with worse outcomes). Consequently, our research question could only be answered by comparing different models or components of health visitors relative to each other, in terms of how they affect outcomes. By describing important differences in health visitors and mapping current models of provision across England, our study will be foundational for a future quasi-experimental study that can take advantage of changes over time within areas and differences across local areas.

## Methods and analysis

### Study design and objectives

This 4-year interdisciplinary mixed-methods study aims to assess which health visiting models in England are most promising for mitigating the harms of exposure to ACEs. This study started on 1 February 2022 and is expected to end on 31 January 2026. The study objectives are to (1) develop a typology of health visiting service delivery models and (2) examine how different health visiting models are likely to work, for whom and in which contexts. To achieve these objectives, we will integrate findings from analysis of individual-level administrative data, aggregate LA-level data, in-depth case studies (including interviews with mothers), a national online survey of health visiting services, and workshops with stakeholders and experts by experience. We will take an explanatory sequential approach to integrate data, where qualitative data collection is used to challenge and explain findings from the quantitative data.^27^

### Description of data sources

#### Linked individual-level administrative data

The Community Services Data Set (CSDS)^28^ is a deidentified, individual-level administrative dataset that includes records of health visiting contacts which is used to generate cross-sectional, LA-level annual statistics related to health visiting.^29^ We will use CSDS data to describe contact with health visiting services between 1 April 2017 and 31 March 2019 for all children in England and develop a typology of health visiting service delivery models. We use CSDS data from LAs, which meet our data quality parameters, described elsewhere.^30^

Hospital Episodes Statistics (HES) is a deidentified, individual-level dataset that contains details of all National Health Service (NHS) hospital admissions in England.^31^ Based on records of births and deliveries in HES, we will identify the cohort of children born in England between 1 April 2015 and 31 March 2019. Using well-established methods, we then will link this cohort to their mothers’ hospital records^23^ and identify mothers who had a hospital admission before their baby was born, which recorded alcohol/substance misuse, domestic violence or mental health issues^26^ (ie, the study definition of ACEs exposure). Although fathers play a key role in parenting and family well-being, our study focuses only on ACEs recorded for mothers for methodological reasons as it is not currently possible to link fathers to their babies within hospital or health visitors records.^32^ This study includes a workshop with fathers to ensure we have a mechanism for taking their perspectives into account.

We will use a pseudonymised linkage key provided by NHS Digital to link deidentified extracts of CSDS and HES data. We will then use this novel, linked data source to assess any differences in health visiting service models, according to exposure to ACEs. We will also use the linked CSDS-HES data to examine differences in health outcomes for children and mothers for up to 5 years after birth.

#### Aggregate LA-level data

We will identify relevant, publicly available, aggregate data to describe the local context in which different models of health visiting delivery operate. For example, this may include LA-level indicators related to local need (eg, deprivation), expenditure on local services and information on how health visitors integrates with other local services (eg, targeted support for teenage mothers through the FNP^13^) available from sources such as public health profiles published by the Office for Health Improvement and Disparities^33^ and local vulnerability profiles published by the Children’s Commissioner.^34^

#### In-depth case studies

We will conduct in-depth case studies of health visitors in up to six LAs comprising interviews with mothers and professionals (eg, health visitors, service managers), documentary analysis and, where available, analysis of locally held administrative data that contains additional information not available in CSDS-HES. These data may include local information on community services and/or GP consultations, hospital services, early help and children’s social care services, adult social care and/or mental health services for carers and parental demographics. Case study sites will be selected to reflect the different models of health visiting service delivery that emerge from our initial typology, as well as the range of rural/urban settings and geographies across England.

Interviews with professionals and mothers (four for each group per site) will be semistructured and audiorecorded. To develop the interview topic guides, we will conduct a literature review of the key principles, functions and mechanisms of effect within health visiting for families with children exposed to ACEs. The interview topic guide for professionals will include prompts related to priorities, constraints and local factors that have shaped health visiting. For example, we will ask health visitors how they identify and work with families at differing levels of need, and how appropriate levels of service are identified, by asking about specific families on their caseload. We will ask mothers about their experiences and perceptions of health visiting, including any out-of-pocket costs associated with accessing the services.

The case studies will be crucial in understanding variation that cannot be detected by the quantitative data alone and will allow us to make sense of our quantitative findings (eg, if we do not detect differences in outcomes between our ‘models’ it might be because our typology and measurable outcomes are not sensitive enough to capture impact).

#### National online survey

The online survey will collect information related to differences in health visiting service delivery that are not captured in CSDS data, including local innovations and targeting guidelines, for example, corporate versus individual caseload models. We will also collect information to measure the costs and resource-use of health visiting service, such as staff type (eg, health visitors or nurses), salary band, capital and non-capital overheads and total expenditure for health visiting service. The survey will be piloted before being circulated to commissioners and other relevant health visitors staff in all LAs in England.

#### Workshops with stakeholders and experts by experience

We will invite up to 40 stakeholders to a half-day workshop to refine our typology of health visiting service delivery models and ensure its relevance to practice. Stakeholders will include public health consultants, health visitors professionals and representatives from the Institute of health visiting, Community Practitioners and Health Visitors Association, Royal College of Nursing, FNP and the Department of Health and Social Care (DHSC). We will also run four workshops with experts by experience to gain views about the acceptability and meaning of the different types of health visitors for parents, including one for fathers.

### Analysis

#### Developing a typology of health visiting service delivery models

**Figure 1:**
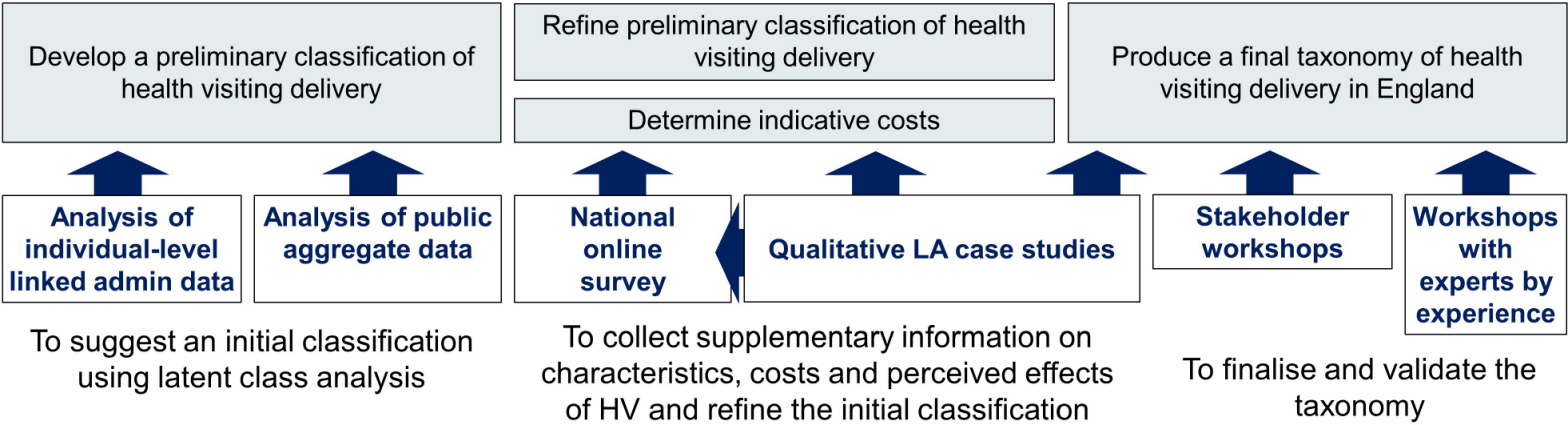
Overview of data sources and approach for developing a typology of health visiting service delivery. LA, local authority.

We will use an empirical-to-conceptual approach when developing our typology of health visiting service delivery in England (figure 1). We will start with a data-driven classification generated from latent class analysis of indicators of health visiting service characteristics derived from CSDS-HES data. We will then deductively conceptualise the nature of each model of health visiting service delivery and refine our classification based on all other available data (ie, aggregate LA-level data, case study data, national survey and consultations with stakeholders and experts by experience). By combining results from these different sources, we will create a final typology of health visiting service delivery with rich descriptions of each model, including associated costs, local context and perceived drivers, consequences, barriers and facilitators. Data will be analysed using Stata version MP17.0 and Nvivo, as appropriate. A detailed statistical plan will be written prior to any quantitative analyses.

#### Latent class analysis of administrative data

Latent class analysis is used to identify hidden, underlying groups in a population based on observable characteristics, known as indicators.^35^ We will derive a range of theoretically relevant indicators of LA-level health visiting service delivery from CSDS-HES data (eg, the proportion of the population receiving mandated visits as an indicator of service reach). The choice of which indicators to include in the latent class model will be guided by theory and a statistical framework for indicator selection.^36^ Similarly, the optimal number of latent classes (ie, models of health visiting service delivery) will be selected based on statistical fit indices^37^ and the interpretability of the classes.

#### Analysis of aggregate LA-level data and case study data

We will carry out descriptive analysis of publicly available, LA-level data (as well as locally held administrative data from case study sites) to understand the local context in which different models of health visiting service delivery operate and to supplement our findings on how LA-level factors and local needs drive differences in health visiting models. This may include describing indicators related to local need (eg, deprivation), expenditure on local services and information on how health visiting integrates with other local services (eg, availability of targeted support for teenage mothers through the FNP).

For interview and documentary data collected in case study sites, we will use thematic analysis to identify common and recurring themes within and across case study sites. We will use quality assurance techniques of simultaneous data collection and analysis, open coding of data to generate new ideas and develop the initial coding framework, and constant comparison between cases looking for negative (‘deviant’) cases to expand and test emerging theory.^38^ Findings that emerge from this analysis will be used to refine the initial classification of models of health visiting service delivery from the latent class analysis.

#### Analysis of national survey data

We will analyse data collected in the national online survey to provide further descriptive information about the factors influencing the intensity and targeting of health visiting services within each model of health visiting service delivery (eg, staff type, local innovation or targeting guidelines).

We will also use information related to costs and resource-use collected in the survey to describe the costs of each model of health visiting service delivery.

#### Findings from workshops

We will present the revised typology at a stakeholder workshop for refinements and validation. Attendees will discuss the extent to which the models of health visiting services that we have proposed reflect their experiences of the service they deliver. We will also derive evidence on the acceptability and meaning of the different models of health visiting from workshops with experts by experience.

#### Examining the association between health visitors models and outcomes for children and mothers exposed to ACEs

We will examine the association between the different models of health visiting service delivery included in our final typology and child and maternal outcomes that (1) can be identified in individual-level CSDS-HES data and (2) are theorised as amenable to intervention by health visiting services. For example, reducing hospital attendance and admissions for injury (through managing minor illnesses and accidents) is one of the six high-impact areas for health visitors in policy guidance^39^; therefore, one of the outcomes we will include is injury-related hospital admissions for children (table 1).

We will analyse CSDS-HES data for all children born in England between 1 April 2015 and 31 March 2020 (approximately 3 million individuals) and their mothers in LAs which meet our CSDS data quality parameters, described elsewhere.^30^ We will model the risk of outcomes using generalised linear models, adjusting for relevant predictors of both ACEs and outcomes, and accounting for clustering within LAs. We will assess model fit using resampling methods, such as bootstrapping. We will stratify our analyses by ACEs exposure to explore which models of health visiting service delivery are most promising for mitigating their harms. To determine whether specific models of health visiting work differently according to local context, we will include interaction terms for LA-level indicators of local area need that measure the wider determinants of health, such as measures of deprivation, ethnic distribution of population or the prevalence of teenage pregnancies. A detailed statistical plan will be written prior to analyses. Data will be analysed in Stata. Study results will be reported in accordance with GUILD,^40^ Strengthening the Reporting of Observational Studies in Epidemiology^41^ and RECORD guidelines.^42^

### Limitations

CSDS is a relatively new data resource for research related to health visiting and there are likely to be issues with data quality. Building on previous work, we will examine the quality of CSDS data with reference to publicly available statistics (such as the health visiting service Delivery Metrics^29^) to identify LAs with data of sufficient quality to be included in the analysis.^30^

A further limitation of this study is that by relying on information recorded during hospital admissions to define exposure to ACEs, we will only capture the severe end of the spectrum. Individuals with exposure to ACEs that were not severe enough to result in and be recorded as part of hospital admission will be misclassified as not being exposed. Based on previous work describing the overlap of patients with ACEs recorded in hospital and GP records, we estimate that we will be able to identify 38% of all ACEs known to health services using HES data.^43^ Although we will be able to identify a significant proportion of children exposed to ACEs, there will be issues with misclassification whereby the ‘non-ACEs’ comparison group will also include those with exposure to ACEs that were not recorded in hospital admissions. This means that any associations that we identify between models of health visiting service delivery and child and maternal outcomes may be underestimates of the true difference. To explore the effect of our study definition of exposure to ACEs, we will conduct sensitivity analyses to look at outcomes for other groups who are likely to be vulnerable to poor outcomes, such as those living in deprived areas or teenage mothers.

The range of outcomes that can be included in our analysis is also a limitation. For example, parent–child interaction, sensitive parenting, parental self-efficacy and stimulation from the home environment are relevant outcomes for evaluating the impact of health visiting service delivery models and are particularly relevant for mitigating the impact of exposure to ACEs. The relationship between parents and health visitors will be central to promoting these outcomes for children and we will explore this in the qualitative component. However, these outcomes are not routinely collected in administrative datasets and for population-level capture would need to be collected directly from parents, which is difficult and expensive. We also cannot evaluate the impact of different health visiting service delivery models on health outcomes that do not meet the threshold for hospital admission as it is not currently possible to link CSDS data to GP records or other administrative health data sources at a national level. Nonetheless, our focus on outcomes that are considered amenable to health Vvisiting which can be identified readily in hospital admissions data will provide the first indication of which health visiting models are most promising for mitigating the impact of exposure to ACEs and will serve as the foundation for future evaluations.

### Patient and public involvement

Our study steering committee, which will be consulted throughout the study period, includes a mother with lived experience of exposure to relevant ACEs. We will run four workshops with four different groups of experts by experience, including one group for fathers, in year 2 of the study. We will seek views on how far the models of health visiting in our typology are acceptable to parents and how they do or don’t reflect parents’ experiences of health visiting. Participants of the expert by experience workshops will also have the opportunity to contribute to the interpretation of results, or coproduce outputs and support the dissemination of the study findings.

## Ethics and dissemination

### Ethics

This study has been approved by University College London Institute of Education (UCL IOE) Research Ethics Committee (1531). We will seek additional approvals from NHS Research Ethics Committee, NHS Trust Research and Development Teams and LA Research and Development Teams for the interviews with mothers and professionals and any analysis of locally held administrative data in the case study sites. Approvals for the use of CSDS linked with HES data have been granted by NHS Digital’s Independent Group Advising on the Release of Data. Our study involves secondary analysis of non-identifiable administrative health data of up to 3 million child–mother pairs who have not provided informed consent. It would not be feasible to obtain consent because we do not have access to names and addresses with which to contact individuals, obtaining these contact details would require further disclosure of personal information and this is a very large number of families. For the survey of professionals and in-depth interviews with parents and professionals, we will gain informed consent from each participating individual.

## Dissemination

The main output from this study will be a detailed typology of health visiting service delivery models in England, including information about which models are likely to be most promising for mitigating the harms of exposure to ACEs. We will contextualise the study findings for and with input from key stakeholder groups. For example, we will work with our parent representatives to identify relevant streams of communication and to coproduce outputs for families including blogs and lay summaries (eg, fact sheets about the role of health visitors from parent perspectives).

Tailored briefings will be disseminated to policy-makers, local directors of public health, commissioners and managers of health visiting service and health visitors, and will provide evidence to inform decisions and directives about how to support families with children exposed to ACEs within a service that remains universal. These findings will be of direct benefit to the health and social care sector by providing detailed and up-to-date evidence to inform decisions. At a national level, the results will inform discussions at DHSC about modernising the Healthy Child Programme, a policy initiative which will be carried out over the coming years, as well as about potential changes to the commissioning and delivery of health visitoring (eg, what is feasible in terms of a minimum number and type of health visitor contacts for high need families). At a local level, our findings will inform decisions about how to run, commission or manage health visiting in the current context of austerity and workforce shortages.

For academic beneficiaries and other researchers, we will aim to publish our findings in high-quality, peer-reviewed journal articles, as well as present at key conferences. Secondary outputs will include methodological research on the quality of the CSDS as a data source for evaluating health visiting and other community services to inform data providers and other researchers on the use of these data for future or ongoing studies.

## Supplementary Material

Reviewer comments

Author's manuscript
