# Supplementary material for: Study protocol: a mixed-methods study to evaluate which health visiting models in England are most promising for mitigating the harms of adverse childhood experiences
Source: BMJ Open. 2022 Sep 28;12(9):e066880. doi: 10.1136/bmjopen-2022-066880 (PMC9528572; doi:10.1136/bmjopen-2022-066880)
Supplement: Reviewer comments [file bmjopen-2022-066880.reviewer_comments.pdf]

PEER REVIEW HISTORY

BMJ Open publishes all reviews undertaken for accepted manuscripts. Reviewers are asked to complete a checklist review form (**http://bmjopen.bmj.com/site/about/resources/checklist.pdf**) and are provided with free text boxes to elaborate on their assessment. These free text comments are reproduced below.

ARTICLE DETAILS

**TITLE (PROVISIONAL)**	Study protocol: a mixed-methods study to evaluate which health visiting models in England are most promising for mitigating the harms of adverse childhood experiences
**AUTHORS**	Woodman, Jenny; Mc Grath-Lone, Louise; Clery, Amanda; Weatherly, Helen; Jankovic, Dina; Appleton, J; Kirman, Jennifer; Barlow, Jane; Kendall, Sally; Bennett, Samantha; Gilbert, Ruth; Harron, Katie

This article was not externally reviewed at BMJ Open. Protocols that have been independently assessed prior to submission to BMJ Open are usually fast-tracked to publication on the grounds that further substantial changes will not be possible. This independent assessment will usually be external, independent review for both a substantial grant award from a non-commercial or government funder and ethics approval.
